# Resilience strengthening in youth with a chronic medical condition: a randomized controlled feasibility trial of a combined app and coaching program

**DOI:** 10.1007/s00787-024-02395-w

**Published:** 2024-03-02

**Authors:** Anne Christine Bischops, L. Sieper, J. Dukart, N. K. Schaal, C. Reinauer, P. T. Oommen, C. Tomoiaga, O. David, E. Mayatepek, T. Meissner

**Affiliations:** 1 Department of General Pediatrics, Neonatology and Pediatric Cardiology, Medical Faculty and University Hospital Düsseldorf, Heinrich Heine University, Moorenstrasse 5, 40225 Düsseldorf, Germany; 2 Institute for Neurosciences and Medicine: Brain and Behavior (INM-7), Research Center Jülich, Jülich, Germany; 3 Institute of Systems Neuroscience, Medical Faculty, Heinrich Heine University, Düsseldorf, Germany; 4 Department of Experimental Psychology, Heinrich Heine University, Düsseldorf, Germany; 5 Division of Pediatric Rheumatology, Department of Pediatric Oncology, Hematology and Clinical Immunology, Medical Faculty, University Hospital Düsseldorf, Heinrich Heine University, Düsseldorf, Germany; 6 International Institute for the Advanced Studies of Psychotherapy and Applied Mental Health, Babes-Bolyai University, Cluj-Napoca, Romania

**Keywords:** Resilience, Youth, Adolescents, Chronic medical condition, Mental health

## Abstract

Youth with a chronic medical condition (CMC) are often affected by comorbid mental disorders. Resilience-strengthening interventions can protect youth’s mental health, yet evidence-based programs remain scarce. To address this lack, this study aimed to evaluate the feasibility of a dual approach combining app-based resilience training and cognitive behavioral group coaching. Fifty-one youths with CMC treated at a German university children’s hospital aged 12–16 years were recruited. They were randomly assigned to a combined app game and coaching intervention or sole app gameplay. At pre-, post-intervention, and at a 2-month follow-up resilience, automatic negative thoughts and an app and coaching evaluation were assessed. Feasibility was defined as a recruitment rate of 70%, an 85% adherence rate for the REThink game, and 70% participation in both coaching sessions. Feasibility criteria were reached for coaching participation but not for recruitment or app adherence. While both the REThink game app and coaching intervention had high acceptance rates among youth with CMC, participants receiving additional coaching sessions showed higher satisfaction and adherence rates. Participants preferred remote to in-person meetings. The findings support a combination of a gamification app approach with online group coaching. Group coaching can improve adherence while online options increase accessibility. Future research should focus on testing in diverse participant samples, language, and age-adapted updates of the REThink game app. These findings provide guidance for increasing adherence in future intervention studies in youth with CMC cohorts.

**Supplementary Information:**

The online version contains supplementary material available at 10.1007/s00787-024-02395-w.

## Introduction

Chronic medical conditions (CMC) affect between 10–12% of youth worldwide today [1]. This alarming number will continue to grow as many conditions show an increasing incidence and improved survival rates. [2, 3] Youth with CMC face substantial challenges in their daily lives and are frequently affected by comorbid mental disorders [4]. Up to 30% of youth with type 1 diabetes have an additional diagnosis of depression or show symptoms of an anxiety disorder [4, 5]. Studies with cancer, inflammatory bowel disease or asthma patients show similar results [6–8].

Resilience generally represents a person’s ability to withstand adversity and successfully adapt to disturbances threatening the person’s function or development [7]. Resilience is essential for coping with everyday life challenges and overcoming adverse life circumstances [9, 10]. Resilient youth show better development, increased well-being, and improved mental health [11, 12]. These resilience skills can be practiced in resilience-promoting interventions [10, 13]. Programs range from school-level curricula and family-level programs to individual interventions. Despite the proven effectiveness of some resilience trainings in healthy youth, interventions focusing on youths with CMC remain scarce and show limited efficacy.[14, 15]

Among the best-proven resilience trainings are group cognitive behavioral therapy (CBT) interventions, with a dosage of eight to twelve sessions typically resulting in optimal results [16, 17]. Youth with CMC are known to prefer briefer interventions for better adherence [18]. However, these short interventions may not provide sufficient dosage to achieve resilience improvement [18]. Sample sizes are mostly small and often lack the power to detect moderate effect sizes [15]. Due to the broad field of CMC, participants are heterogeneous and may face illness-specific challenges [15]. In addition, studies often encounter major recruitment difficulties [18, 19]. Accessibility and time consumption are high barriers for youth already struggling with a chronic disease [19]. Youth tend to underutilize mental health services such as resilience trainings due to a desire of autonomy and fear of stigmatization [20]. Engaging and age-appropriate content is, therefore, crucial to reach this target group which is characterized by poor adherence and low resources [20].

A possible solution for improved accessibility and attractiveness is digital interventions, especially in this age group with ubiquitous smartphone use [21]. Online interventions can reduce stigma, decrease costs, address staff shortages, and offer more flexible, self-controlled scheduling—all core issues for youth with CMC [22]. The recent increase in mental health apps offers numerous ways of psychological support; however, most of these apps lack evidence and are restricted to adult users [23]. Some resilience-building apps specifically for youth are currently being developed or evaluated [24–26]. The “REThink” app is a promising solution that was specifically designed to improve the emotional resilience of youth and had a significant effect on emotional symptoms and emotion regulation [27, 28]. Its value in youth with CMC yet remains to be demonstrated. Considering the long-term experience with face to face CBT trainings, the combination of a gamified app with CBT sessions seems promising [29]. While the gamified app format can address previous limitations of accessibility, attractivity and time consumption, the CBT sessions can deepen resilience-strengthening contents. Indeed, additional human guidance has shown to be a beneficial feature for digital interventions resulting in stronger intervention effects [14, 30, 31]. Concerning the major recruitment issues of previous in-person trainings, blended approaches of digital offers with additional synchronous coaching could improve participation and motivation while also providing a feeling of support [30, 32]. To our knowledge, no similar study has yet been conducted for youth with CMC. To address the current lack of resilience-building interventions for youth with CMC, this study aimed to evaluate the feasibility of a combined group coaching and app approach through the following research questions:

- 1. Can youth with CMC be motivated to participate in a combined app game and coaching format?
- Hypothesis 1: 70% of youth who meet the inclusion criteria can be recruited.
- 2. Is the REThink app game accepted by youth with CMC?
- Hypothesis 2: 85% of participants play all levels of the REThink app game.
- 3. Is the group coaching format accepted by youth with CMC?
- Hypothesis 3: 70% of participants in the REThink + Coaching group participate in both group sessions.

As previous studies have reported high acceptance for remote group interventions, the effect of a remote coaching option on adherence was also evaluated [33].

## Methods

### Participants and setting

Youth with CMC were recruited between April and July 2022 upon presentation at the University Children’s Hospital Duesseldorf, Germany. Inclusion criteria were (1) age between 12 and 16 years, (2) having a CMC (defined as having a disease for over a year, mandatory long-term medical care or a significant impairment of daily life routine), (3) sufficient knowledge of German or English for app usage and coaching participation, (4) sufficient physical and mental condition for study participation (Lansky score ≥ 80 [34]), (5) access to an Internet-enabled smart phone, (6) a signed informed consent form by both parents and participants, (7) no prior history of psychiatric or psychological treatment in the last 3 months or longer than 3 months). Based on the approach of Whitehead et al., at least 25 participants per group were planned for a subsequent main trial designed with 90% power and an effect size of 0.1 ≤ δ < 0.3 (Cohen’s d) [35]. The minimum sample size, therefore, was *n* = 50.

### Outcome measures

Primary endpoint was the feasibility of the REThink + Coaching intervention.

The feasibility criteria (as detailed in the Introduction section) were based on previous study results [28, 36].

Based on the principles of Thabane et al. [37], the following secondary outcomes were evaluated:

- What is the youths’ adherence (rejection rate, loss to follow-up rate)?
- What are reasons for participation/non-participation?
- What do youth like and dislike about the app and coaching?
- Which population groups does the study reach?
- Considering the overall population of youth with CMC, should one offer a low-threshold resilience app game or a combined training intervention with in-depth coaching?

In addition, resilience and automatic negative thoughts were examined at baseline, post-intervention, and in 2-month follow-up.

### Sociodemographic questionnaire for youth and parents

The sociodemographic questionnaire comprised information on age, gender, nationality, chronic disease, educational attainment, socioeconomic status, media and app use. Subjective socioeconomic status was assessed as described by Lampert et al. in the KiGGS Wave 2 study [38].

### Coaching and app game evaluation questionnaire

Usefulness and satisfaction were assessed on a five-point Likert scale for app and coaching. Based on the questionnaire by David et al. usage difficulties, target age group, liked or disliked aspects, preferred app level, subjective resilience improvement, recommendation for friends and preference of in-person or online meetings were assessed for app and coaching if applicable [39].

### Resilience Scale 13 (RS-13)

The 13-item Resilience Scale (RS-13) is the short form of the Resilience Scale (RS-25) by Wagnild et al. and measures resilience as a person’s positive characteristic of individual adaptability [40]. Participants are asked to indicate their agreement with certain phrases on a seven-point Likert scale. The RS-13 has high internal reliability (Cronbach’s *α* = 0.90), the retest reliability is good (0.62) [40]. A sum score from 13 to 66 points is considered as low resilience, 67 to 72 points as moderate, and 73 to 91 points as high resilience [40].

### Children’s Automatic Thoughts Scale (CATS)

The Children’s Automatic Thoughts Scale is a 40-item questionnaire that assesses negative self-statements in children and adolescents [41]. It comprises four subscales: physical threat, social threat, personal failure, hostility. Participants rate to what extent they had the respective thought over the past week on a five-point Likert scale. The CATS showed high internal consistency (Cronbach’s* α* = 0.95), the retest reliability was good (0.76) [41]. A sum score of ≥ 70 points is considered as clinical cut-off point for internalizing or externalizing problems (anxiety, depression or behavior disorder).

### Study design

In a two-arm randomized controlled trial (RCT), participants were randomly assigned to either the REThink + Coaching or only REThink app group (stratified by age and gender). If participation was refused, youths and parents were asked to state their reasons for rejection anonymously. Eligible youths were identified through search of the specialty clinics’ patient registries and the emergency room and inpatient calendars. Before enrollment, a random allocation sequence list was generated using GraphPad Prism by an independent study team member [42]. The allocation sequence list was stored in a sealed folder and consulted for allocation after successful recruitment. Participants and study team members were not blinded due to obvious difference of the interventions.

Youth participants completed the RS-13 and CATS pre-, post-intervention (defined as 7 weeks after baseline) and in a 2-month follow-up. At baseline, youth and parents further completed a sociodemographic questionnaire. Post-intervention youth also received an evaluation questionnaire and participated in a short semi-structured interview on app and coaching evaluation. Participants were reminded a maximum of three times via E-mail to complete the questionnaires. The participants received a total of 20 Euro in gift vouchers as incentives. Detailed information on study procedures and data collection can be found in the Supplementary methods.

### Intervention

#### REThink game app

The REThink game app was developed by David and collaborators to promote emotional well-being and resilience in youth. Based on Rational Emotive Behavioral Therapy, the REThink game app focuses on teaching youth emotion regulation and coping strategies for dysfunctional negative emotions, through seven levels [39]. Detailed information on app content and development can be found in the supplementary methods.

For this study, an English audio version with German subtitles was developed. The player’s date of play and level played were collected. Participants were instructed to complete one level every week for 7 weeks based on the validation study protocol. If the corresponding module of the week had not been played, participants were reminded via E-mail (once per week).

#### Cognitive behavioral therapy-based online coaching

The coaching manual was jointly developed by the study team and pediatric psychologists. The coaching content was based on cognitive behavioral therapy and coordinated with the app topics. The coaching sessions were led by experienced clinicians and psychology students with a bachelor’s degree. Detailed information on manual development and session content can be found in the supplementary methods.

REThink + Coaching participants were instructed to play the REThink app and received two additional group coaching sessions in groups of 6–10 participants (duration 60–90 min). They could choose between online or in-person sessions at the children’s clinic; however, no in-person coaching sessions were held due to low demand. Participants were reminded to participate via E-mail or phone 1 day before their sessions.

### Statistical analysis

The analysis focused on a quantitative evaluation of feasibility. However, data on the necessary parameters for use in a larger future RCT were collected. Data from all randomized participants were analyzed (intention-to-treat-collective). First, a descriptive analysis of the sociodemographic data, RS-13/CATS scores at baseline and evaluation forms was performed. Socioeconomic status quintiles were calculated according to Lampert et al.[38]. Chi-square tests/*t *tests were used to determine if there were significant differences by group at baseline (for frequencies < 5 fisher’s exact test was used). For the analysis of differences by group between baseline, post-intervention, and follow-up, normality tests (Shapiro–Wilk test) revealed not-normally distributed differences for the difference between group B RS-13/CATS scores pre-intervention and at follow-up. Thus, Wilcoxon signed-rank test was used (see Supplementary Table 2). Third, an exploratory analysis using ANOVA calculations was performed to test for group x time interaction effects. The effect size for mean differences between groups was estimated according to Morris [43, 44]. Univariate and multivariate logistic regressions were computed for coaching and app adherence with age, disease, socioeconomic status, and group membership as explanatory variables. Analyses were performed in SPSS version 28.0. and R version 4.2.1, and figures were created using Adobe Illustrator CC 2019 [45–47]. The study findings are reported according to the CONSORT statement and the Cochrane risk-of bias tool for randomized trials (RoB 2) (see Supplementary Information for checklists).

## Results

### Recruitment and attrition

**Fig. 1:**
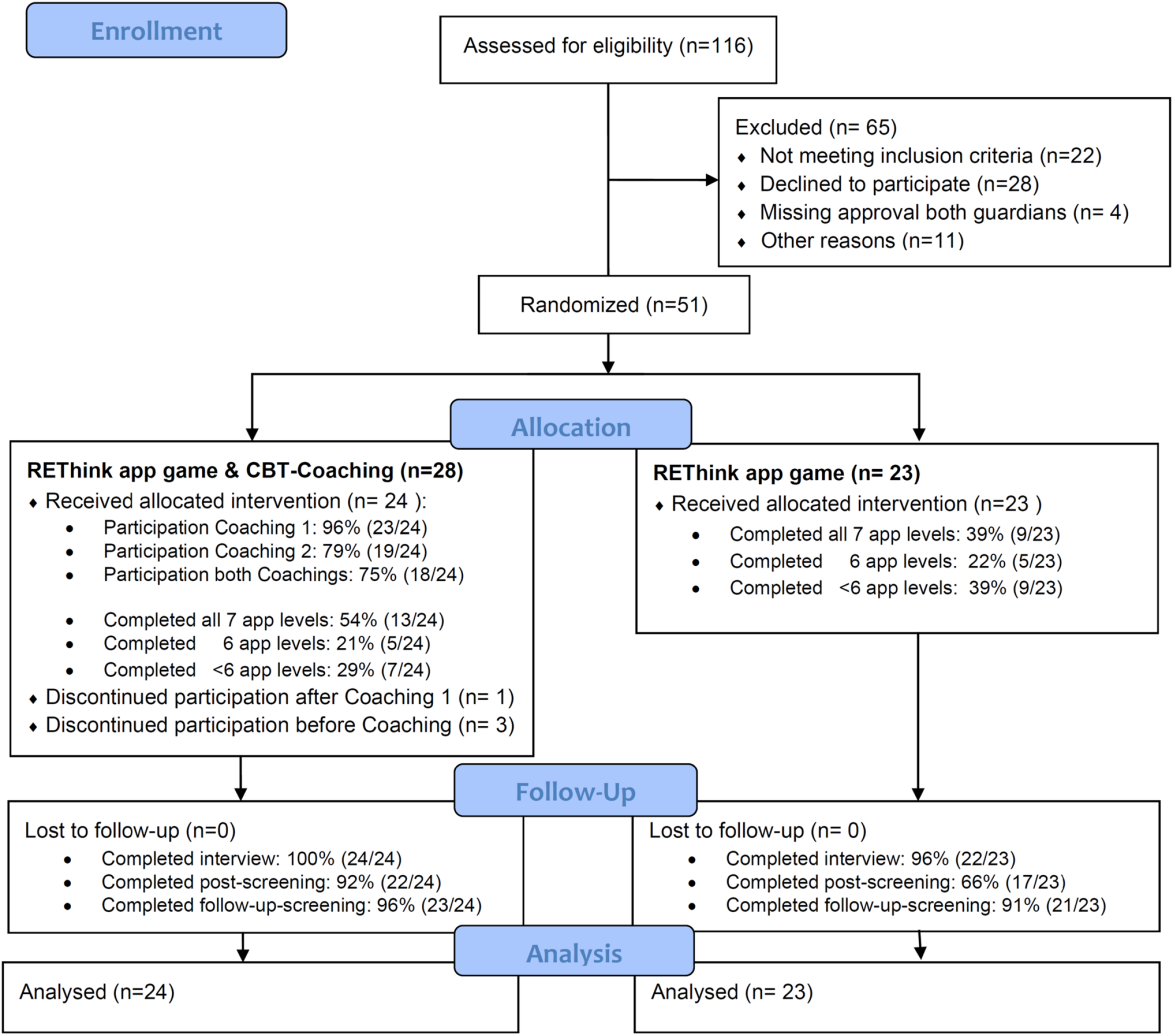
CONSORT flow diagram of study participants

Figure 1 depicts the participants flow through each study phase [48]. Fifty-one of ninety-four eligible youths were recruited (54.3%). The main reason for participation refusal was time constraints, followed by a lack of interest in the topic, general lack of meaningfulness of study participation, and too low incentives (see Supplementary Fig. 1). A lack of language proficiency and time constraints were the main barriers for parental study participation. A total of 47 youths with CMC received the allocated intervention (see Fig. 1). All but one youth completed the post-intervention interview, 94% completed the 2-month follow-up screening.

In the multivariate regression model, a higher socioeconomic status was associated with an increased probability of coaching completion. App adherence was not predicted by disease, group membership or socioeconomic status. However, age was a strong predictor for app adherence (see Supplementary Table 3).

### Sample characteristics

Participants were on average 14.2 years old, 60% identified as female (see Table 1). 57% had type 1 diabetes mellitus, the second most common disease was Crohn’s disease (11%). All participants lived in households with a medium or high socioeconomic status. Chi-square and *t* test analyses indicated no difference in baseline characteristics which suggests successful randomization (see Table 1).

**Table 1:** Sample characteristics

	Total	REThink	REThink + Coaching	*t test/Chi-square (df)*	*p value*
*n*	47	23	24	–	–
Gender, *n* (%)				1.93 (2)	0.37
Female	28 (59.6)	15 (65.2)	13 (54.2)		
Male	18 (38.3)	7 (30.4)	11 (45.8)		
Other	1 (2.1)	1 (4.3)	0 (0.0)		
Age, *n* (%)				−0.38	0.70
12	9 (19.1)	5 (21.7)	4 (16.7)		
13	8 (17.0)	5 (21.7)	3 (12.5)		
14	6 (12.8)	2 (8.7)	4 (16.7)		
15	14 (29.8)	5 (21.7)	9 (37.5)		
16	10 (21.3)	6 (26.1)	4 (16.7)		
RS-13 score at baseline				−0.19	−0.696
Mean (SD)	66 (12.2)	65.7 (12.1)	66.4 (12.6)		
Median (IQR)	68 (15)	69 (14)	68 (16)		
Missings	1 (2.1)	0 (0.0)	1 (4.2)	-	-
CATS score at baseline					
Mean (SD)	67.0 (21.0)	70.3 (20.2)	63.8 (21.8)	1.05	0.30
Median (IQR)	63 (27)	73 (32)	57 (17)	1.06	0.30
*Missings*	1 (2.1)	0 (0.0)	1 (4.2)	–	–
Disease^a^, *n* (%)				25.59(24)	0.27
Type 1 diabetes	26 (54.9)	15 (65.2)	11 (45.8)		
Inflammatory bowel disease	6 (12.7)	1 (4.3)	5 (20.9)		
Inflammatory rheumatic disease	5 (10.5)	3 (12.9)	2 (8.4)		
Thyroid disease	3 (6.3)	2 (8.6)	1 (4.2)		
Other^b^	6 (12.7)	2 (8.6)	4 (16.8)		
Missings	1 (2.1)	0 (0.0)	1 (2.1)	–	–
Type of School, *n* (%)				2.88(4)	0.70
“Hauptschule“	1 (2.1)	1 (4.3)	0 (0.0)		
“Realschule“	10 (21.3)	6 (26.1)	4 (16.7)		
“Gymnasium“	23 (48.9)	10 (43.5)	13 (54.2)		
“Gesamtschule“	11 (23.4)	5 (21.7)	6 (25.0)		
Vocational college	1 (2.1)	1 (4.3)	0 (0.0)		
Missings	1 (2.1)	0 (0.0)	1 (4.2)	–	–
Socioeconomic status, *n* (%)				0.03 (37)	0.97
Quintile 1 (low)	0 (0.0)	0 (0.0)	0 (0.0)		
Quintile 2 (medium)	1 (2.1)	0 (0.0)	1 (4.2)		
Quintile 3 (medium)	11 (23.4)	6 (26.1)	5 (20.8)		
Quintile 4 (medium)	16 (34.0)	8 (34.8)	8 (33.3)		
Quintile 5 (high)	11 (23.4)	6 (26.1)	5 (20.8)		
Missings	8 (17.0)	3 (13.0)	5 (20.8)	–	–
Nationality, *n* (%)				0.36 (1)	0.55
German	43 (91.5)	21 (91.3)	22 (91.7)		
Other	3 (6.4)	2 (8.7)	1 (4.2)		
Missings	1 (2.1)	0 (0.0)	1 (4.2)	–	–

*n* number*, Type 1 DM* type 1 diabetes*, SD* standard deviation

^a^ Primary treatment diagnosis

^b^ Other diseases mentioned: hyperinsulinism, myocarditis, celiac disease, neurofibromatosis, pseudotumor cerebri, biotinidase deficiency

### Acceptance and evaluation of REThink app

45.8% completed all levels of the REThink app and 20.8% played at least six levels. Out of the 16 youths who played less than 6 levels or did not play at all, 94% still participated in the post-intervention assessment.

**Fig. 2:**
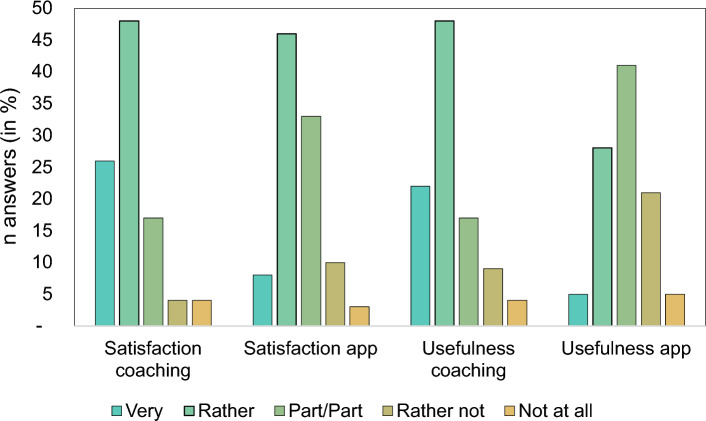
Participant’s opinion on usefulness and satisfaction of coaching and REThink app game. Answers n = 39 for app evaluation, n = 23 for coaching evaluation

39% of the REThink group and 52% of the REThink + Coaching group completed all levels. 54% indicated to be very or rather satisfied with the app, 33% considered the app as very or rather useful (see Fig. 2).

**Fig. 3:**
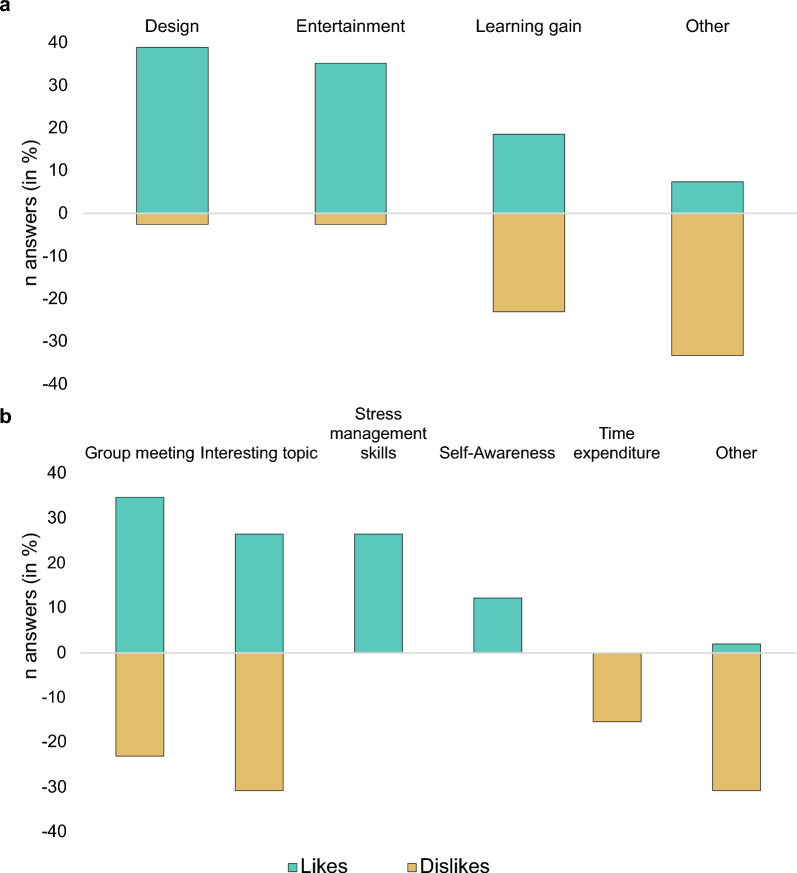
Likes and dislikes of **a** REThink app and **b** coaching intervention. Multiple answers possible, percentage in relation to total number of mention (*n* app likes/dislikes = 39, n coaching benefits = 49, *n* coaching downsides = 26). Number of answers inverted for “Dislikes” for better visualization. Other: likes—explanations, repetition of levels, dislikes—for younger age group, music annoying, level too complicated/too easy, not enough time, not enough levels, coaching likes—coaching format, group coach, coaching dislikes—not enough games, group too small, English examples, camera use, length, not everyone talking

41% felt a subjective improvement in their resilience after usage. 69% of the participants felt the app was easy to use and would recommend it for youth aged < 12 years (see Supplementary Tables 4 and 5). 8% reported difficulties with usage, the main reason being English audio instructions. Participants especially appreciated the design and entertainment factor. 18.5% perceived a learning gain, while 23.1% reported no perceived benefit (see Fig. 3).

Detailed information on reasons for difficulties of app usage, general app or video game preferences, and data on media use can be found in the appendix (see Supplementary Tables 6–12 and Supplementary Figs. 2–3).

### Acceptance and evaluation of coaching intervention

88% participated in the first coaching session (22/25), 79% (19/24) participated in the second. Overall, 72% (18/25) of youths participated in both sessions. Three participants of the REThink + Coaching group discontinued participation before intervention, one participant after the first coaching session. 67% received both coaching within 21 days, the maximum duration between the two coaching was 90 days. Reasons for postponement were sickness, school holidays, and home repairs. 74% of youths were very or rather satisfied with the coaching, 70% of the participants considered the coaching as very or rather useful (see Fig. 2).

Figure 3 presents the participant’s perceived benefits and negative aspects of the coaching. Meeting other peers was the most important advantage (34.7% of participant’s answers). 26.5% and 12.2% perceived learning about stress management skills and self-awareness as beneficial, 15.4% reported the time expenditure as negative. 31% were not interested in the study topic. 91% of the coaching participants opted for online meetings and 59% considered the length of 45–60 min appropriate, while 22% would have preferred a shorter duration. Over half of the participants (55%) would have liked to continue with coaching and 41% perceived an improved resilience.

### Resilience and intervention effects

**Fig. 4:**
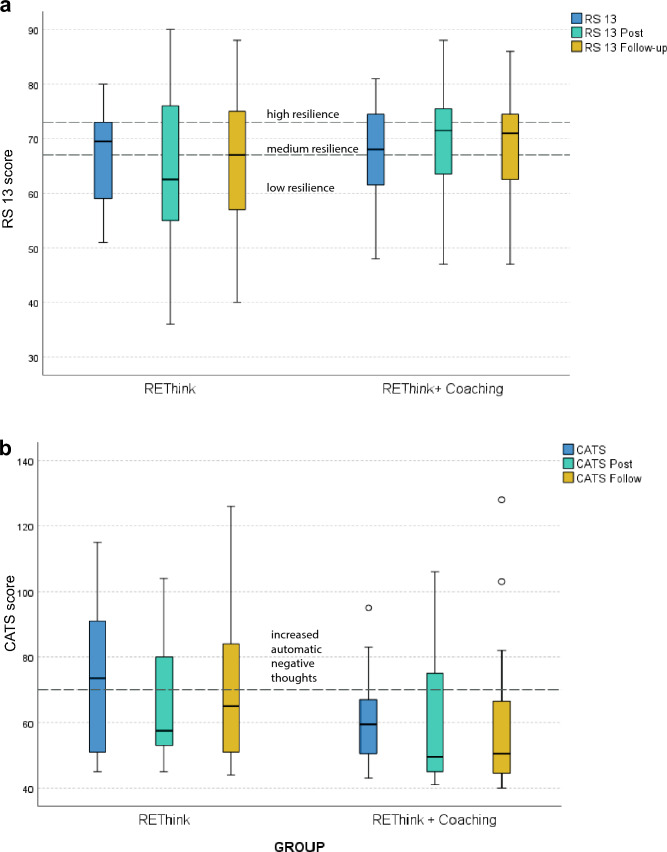
Boxplots with **a **RS-13 and **b** CATS scores at baseline, post-intervention, and 2-month follow-up by group

Participants had on average a baseline RS-13 score of 66 indicating low resilience [40]. There was no significant effect over time (pre/post/follow-up) for RS-13 scores (*F* (2.0, 72.0) = 0.308, *p* = 0.736, partial *η*^2^ = 0.008). Similarly, no significant interaction effects between time and group were found (*F* (2.0, 72.0) = 1.930, *p* = 0.153, partial *η*^2^ = 0.051). The resilience scores by group over time are presented as boxplots in Fig. 4 and Table 2. In the REThink + Coaching group, the RS-13 mean score increased significantly post-intervention and at follow-up, while there was no significant change in the REThink group. Exploratively, the difference between REThink + Coaching and REThink corresponded to a medium effect of *d *= 0.53 [43].

**Table 2:** RS-13 and CATS scores and their mean differences at pre/post/follow-up

*RS-13*							
Group	Mean pre (SD)	Mean post (SD)	Mean follow-up (SD)	*z* pre/post	*p*	*z *pre/post	*p*
REThink	65.7 (12.1)	63.4 15.0)	64.4 (11.3)	0.741	0.459	−0.056	0.955
REThink + Coaching	67.5 (10.1)	70.7 (9.8)	68.8 (10.3)	2.036	0.042	2.000	0.046
CATS							
Group	Mean pre (SD)	Mean post (SD)	*Mean follow-up (SD)*	*z pre/post*	*p*	*z *pre/post	*p*
REThink	70.3 (20.2)	69.4 (19.9)	74.0 (24.6)	0.698	0.458	0.541	0.588
REThink + Coaching	63.8 (21.8)	58.2 (19.0)	60.4 (22.2)	0.967	0.334	0.939	0.348

*p*  *p* value, *z*   *z* score Wilcoxon signed-rank test

### Automatic negative thoughts and intervention effects

On average, participants had a total CATS score of 67.0 (SD 21.0) points at baseline, which is below the clinical cut-off point of 70 indicating internalizing or externalizing problems (the lower the better) [41]. 36% (17/47) had values over 70 points. The CATS scores by group over time are presented as boxplots in Fig. 4 and Table 2 There was a significant effect over time for CATS scores (*F* (2.0,72.0) = 4.21, *p* = 0.023, partial *η*^2^ = 0.105); however, no significant interaction effects between time and group were found (F (2.0,72.0) = 0.614, *p* = 0.544, partial *η*^2^ = 0.017). The effect size estimate of *d* = −0.13 for the difference between both groups is very low.[43].

## Discussion

Here we evaluated the feasibility of resilience interventions in youth with CMC by comparing a combined app and coaching approach to a stand-alone resilience app game. While both the REThink game app and coaching intervention had high acceptance rates among youth with CMC, participants rated the coaching as more useful. The introduction of two additional coaching sessions improved adherence. Participants preferred remote to in-person meetings.

The observed recruitment rate was below the 70% derived from previous studies of the REThink app with healthy youth which is likely due to differences in the target population of youth with CMC [28]. Also, in the aforementioned study, the game was played two times per week which is another variation in the present study. The addition of coaching sessions might have positively influenced the motivation to participate. Overall, our study showed a similar recruitment rate as compared to other clinical trials involving CBT trainings in youth with CMC [18]. In line with previous findings, the main participation barrier was time constraints [19, 49]. Further reasons for refusal to participate were no interest in the topic and too low incentives. This is an important aspect to consider in future study designs as recruitment information might not have addressed the respective age group. Incentives are a key factor for study participation and must be sufficiently adapted. Accessibility is often reported as an important barrier for participation [19]. With all coaching sessions being held remotely, our study supports these findings and could successfully address this issue with online options. Accordingly online meetings are often preferred [50]. The loss to follow-up rate of 7.8% was lower than in similar studies, weekly e-mail reminder and calls prove to be an effective tool [51].

We further evaluated whether the good results of the REThink game app are transferable to youth with CMC. Even though over half of the participants stated satisfaction with the app and liked the design and entertainment factor, the ambitious goal of an 85% adherence rate within 7 weeks was not reached (45.8% adherence rate). An important reason for this might be the app’s language options. Even though the app offered German subtitles, many users disliked the English audio descriptions. On the other hand, 69% perceived the app as easy to use and rather recommended it for youth under 12 years. Adding a native language audio version, testing in younger age groups as well as an adaptation of difficulty with age may be promising research avenues in this regard. While previous studies reported the advantages of a more flexible and self-controlled approach, participants often needed the weekly reminders to complete the app levels [14, 21]. The app-based approach can help reduce costs and personnel efforts when compared to a face to face intervention. Overall, when using digital interventions such as the REThink game app, one has to carefully consider the distinct increase of youth’s media use in recent years, especially during the COVID-19 pandemic [52]. Potential adverse effects on the developing brain, decreased well-being, and reduced self-esteem are important to acknowledge in intervention design [53]. Yet, when tailoring interventions to the youths’ interest, the growing importance of digital media in youth life has to be taken into account. This might even be an opportunity to show them healthier ways of navigating digital media through digital resilience building.

In terms of reducing the mental health stigma, the prospect of “app game testing” instead of starting resilience training may have helped to reduce this stigma.

The feasibility criterion which was reached was the coaching completion rate of over 70%. The REThink + Coaching group did not only have a high adherence rate at the coaching sessions, but also a higher app adherence rate (52% in REThink + Coaching versus 39% in REThink group). Over half of the participants even would have liked to continue the coaching sessions. They were highly satisfied with the coaching and considered it as useful. Meeting peers and interest in the topic were simultaneously the main perceived advantages and disadvantages, which shows that opinions were divided. While peer support is often beneficial and enjoyed by youth with CMC [54], these findings show that a personal group setting can also lead to unease. Group coaching needs to carefully handle this balance between peer pressure, privacy, and fear of stigmatization issues while offering exchange opportunities. Even though an online setting showed to be more practical for most youth, the results underline the benefit and importance of personal contact with peers and coaches in a live group setting. Learning about mental health topics offers divided opinions. While some enjoyed learning about stress management and awareness skills, others were disinterested in the topic. This underlines again the importance of age-adapted, engaging content for increased acceptance.

Only few digital resilience-strengthening interventions exist for youth with CMC and even fewer have obtained positive outcomes [14, 18]. Even though the study was not powered to detect intervention effects, the RS-13 score increased significantly in the REThink + Coaching group in contrast to the REThink group and we found a medium effect size of the difference between groups observed in our study which is suggestive of a coaching effect on participant’s resilience. Also, 41% of both groups perceived a subjective improvement in their resilience. We did not find an effect on automatic negative thoughts which may point to the interpretation that the intervention did not meaningfully affect negative thoughts, or the CATS scale is not sensitive enough to detect changes in the participant’s negative thoughts. The intervention duration may also have been too short for a sufficient change of negative thoughts. On the other hand, 59% of participants preferred the lengths of the coaching, 22% even would have preferred shorter sessions which might again positively affect adherence. These challenges were also reported in previous resilience pilot studies [18, 22]. Our findings provide a solid foundation to improve the design of future larger clinical trials. Future studies should examine these effects in a larger sample or with an intensified coaching program.

Many previous studies reported illness-specific interventions and thus, feasibility varied by disease and effects were difficult to compare across studies [1, 22, 55]. Our study represents a universal approach which offers disease-independent skills training. Concerning disease stigma, participants were addressed as app and coaching testers for other youth with CMC and often did not perceive themselves as diseased. This change of perspective can facilitate discussing sensitive and personal topics with peers [56]. A universal approach further supports a focus on mutual resources instead of disease deficits.

In terms of coverage of the study, all targeted age groups were successfully recruited, and the participants had a balanced gender distribution. Yet, a limitation of the study is the low disease and socioeconomic heterogeneity of the participants. A large proportion of the recruited youths were diabetic patients due to the diabetes department being the clinic’s largest specialty section. The study only reached youth with a medium or high socioeconomic status and good education which is a common finding among studies targeting mental health in youth [57]. Several parent questionnaires which stated a low level of school education or their native language not being German often did not complete questions on profession or further education which may have led to a bias in answers. The inclusion criterion of having access to an internet-enabled smartphone might have added to this recruitment gap. Compared to other studies, our sample had a slightly higher amount of parents with a foreign background and could reach various nationalities [58]. Again, offering native language surveys and coaching might increase this number. Reaching youth from disadvantaged families with low socioeconomic status remains an important challenge.

## Conclusion

In summary, our study supports the feasibility of a combination of gamification approaches with online group coaching for resilience strengthening. While both app and coaching solutions are well accepted among youth with CMC, participants receiving additional coaching sessions show higher satisfaction and adherence rates. The time and willingness of youth with CMC are limited; thus, online options improve accessibility. Key to effective coaching sessions will be to balance the length and content of sessions for retaining high adherence and sufficient input for resilience improvement. Future research may include adaptations for more diverse participant samples, updates of the REThink app game, and larger studies for the detection of intervention effects. Ultimately, this line of research may lead youth with CMC toward higher resilience to better prepare them for their various daily challenges.

## Supplementary Information

Below is the link to the electronic supplementary material.

Supplementary file1 (PDF 400 KB)

## Data Availability

The datasets and analysis data are available from the corresponding author on request.
