# Supplementary material for: Resilience strengthening in youth with a chronic medical condition: a randomized controlled feasibility trial of a combined app and coaching program
Source: Eur Child Adolesc Psychiatry. 2024 Mar 2;33(9):3273–85. doi: 10.1007/s00787-024-02395-w (PMC11424734; doi:10.1007/s00787-024-02395-w)
Supplement: Supplementary file 1 — Supplementary file1 (PDF 400 KB) [file 787_2024_2395_MOESM1_ESM.pdf]

Supplementary Information for

Resilience strengthening in youth with a chronic medical condition- a randomized controlled feasibility trial of a combined app and coaching program

A.C. Bischops[1], L. Sieper[1], J. Dukart[2,3], N. K. Schaal[4], C. Reinauer[1], P.T. Oommen[5], C. Tomoiaga[6], O. David[6], E. Mayatepek[1], T. Meissner[1]

[1] Department of General Pediatrics, Neonatology and Pediatric Cardiology, Medical Faculty and University Hospital Düsseldorf, Heinrich Heine University, Düsseldorf, Germany

[2] Institute for Neurosciences and Medicine: Brain and Behavior (INM-7), Research Center Jülich, Jülich, Germany

[3] Institute of Systems Neuroscience, Medical Faculty, Heinrich Heine University Düsseldorf, Düsseldorf, Germany

[4] Department of Experimental Psychology, Heinrich-Heine-University, Düsseldorf, Germany

[5] Department of Pediatric Oncology, Hematology and Clinical Immunology, Medical Faculty, Division of Pediatric Rheumatology, University Hospital Düsseldorf, Heinrich-Heine-University, Düsseldorf, Germany

[6] Department of Clinical Psychology and Psychotherapy, Babes-Bolyai University, Cluj-Napoca (UBB), Romania

Corresponding author: Dr. A.C. Bischops, Department of General Pediatrics, Neonatology and Pediatric Cardiology, University Hospital Düsseldorf, Moorenstrasse 5, 40225 Düsseldorf, Germany, Tel.:0049-2111817687, E-Mail: annechristine.bischops@med.uni-duesseldorf.de,

Content

Supplementary methods 3

Supplementary analysis data 6

Supplementary data on adherence 7

Supplementary data on app and coaching evaluation 8

Supplementary data on media use 9

CONSORT 2010 checklist of information to include when reporting a pilot or feasibility trial 12

Cochrane risk-of-bias tool for randomized trials (RoB 2) checklist 15

Supplementary methods

Recruitment and data collection

In a two-arm randomized controlled trial (RCT) participants were randomly assigned to either the REThink + Coaching or only REThink app group (stratified by age and gender). Before the enrolment started, a random allocation sequence list was generated with GraphPad Prism by a study team member not involved with enrollment, another study team member enrolled and assigned participants according to the predefined list.[1] If participation was refused, youths and parents were asked to complete a questionnaire stating their reasons for rejection anonymously.

Data were collected either on paper, on tablets or online using ‘SoSci-Survey’, an online survey tool hosted on encoded servers in Germany compliant with GDPR (EU General Data Protection Regulation) or on paper[2]. Eligible youths were identified through a search of the specialty clinics’ patient registries (diabetes clinic, endocrinology clinic, rheumatology clinic, gastroenterology clinic and metabolic disease clinic) and the emergency room and inpatient calendars. The study was advertised as “app game testing with the opportunity to be drawn for additional coaching”. The app game theme of the superhero “RETman” was implemented in the coaching sessions and recruiting material. Youths and parents were contacted at least 24 hours before their scheduled medical appointment to inform them about the study and provide consent, at presentation the study was again explained in detail and written consent was obtained if not already given. Youths presenting in the emergency department without a scheduled appointment were informed about the study in person and given 24 hours to consent. The baseline assessment of the participants and parents and installation of the REThink app was completed while waiting for their appointment or afterwards (duration 30 minutes on average). Recruitment was performed from 4th April to 8th July 2022 and was stopped after reaching the required participant number.

At baseline (directly after enrolment), youth participants completed the RS-13, CATS) and the sociodemographic questionnaire. Parents additionally completed the parental questionnaire. Post-intervention (which was defined as seven weeks after baseline assessment) and two-month post-intervention youths again completed the RS-13 and CATS. Post-intervention they additionally received an evaluation questionnaire and participated in a short semi-structured interview on app and coaching evaluation. Participants were reminded a maximum of three times (once per week) via E-mail to complete the questionnaires.

<u>Intervention</u>

<u>*REThink game app*</u>

Development of REThink game app

The REThink game app was developed byDavid and collaborators, to promote emotional wellbeingin children and adolescents. The app game was developed based on the Rational Emotive Therapy educational curricula.[3] For detailed information on the development process refer to the previous reports.[4-6] The

Romanian version has been evaluated in different randomized controlled trials including 165 healthy youths at a Romanian middle school and children with self-reported maltreatment showing a significant effect on emotion regulation and emotional symptoms.[6-9]For this study the English audio version of the game was supplemented with German subtitles.

REThink game app content

Based on Rational Emotive Behavioral Therapy, the REThink app focuses on teaching youth coping strategies for dysfunctional negative emotions.[5]The game's storyline is to save the world from the "Irrationalizer" who has filled the world with irrational thoughts with the superhero "RETMAN" by helping people to think more rational and be happier while completing seven levels with different mini-games.[5] The first four levels focus on the identification and differentiation of emotional reactions, the connection to behavioral reactions and the changing of irrational perceptions. The other three levels address the development of problem-solving skills, relaxation and happiness skills (see Supplementary Information Table 1).

Supplementary Information Table 1: Objectives of REThink app levels [7]

Level	Objective
1	1. Identifying emotional reactions, differentiating between basic emotions, complex emotions and functional and dysfunctional emotions<br>2. Identifying cognitive processes
2	1. Identifying cognitive processes<br>2. Identifying the relation between cognitive processes, emotions and behavioral reactions
3	1. Identifying the relation between cognitive processes, emotions and behavioral reactions<br>2. Changing irrational cognitions into rational cognitions
4	1. Changing irrational cognitions into rational cognitions<br>2. Building problem-solving skills
5	1. Building problem-solving skills<br>2. Building relaxation skills
6	1. Building relaxation skills<br>2. Consolidation of previous skills and building happiness skills
7	1. Consolidation of previous skills and building happiness skills

App implementation. With the help of a study team member participants installed the app on their smartphone and created a pseudonymized game account. During game play the player's date of play and level played were collected and securely transferred to a server of the Babes-Bolyai University research group.

Participants were instructed to play the REThink app game at least once a week and to complete at least one level every week for seven weeks. If the corresponding module of the week had not been played, participants were reminded via E-mail (once per week).

Cognitive behavioral therapy based online-coaching

Development of coaching manual

The coaching manual was jointly developed by pediatric psychologists, an experienced youth coach and the study group's medical team based on cognitive behavioral therapy. The decision on this intervention format was based on an extensive literature review.[10] The coaching content was coordinated with the app topics.The manual was piloted twice in healthy youths and adapted according to the youth's feedback.

Coaching content

In the first coaching session, participants were introduced to Albert Ellis' "ABC model" and learned about the identification of thoughts, emotions, and the connection to behavioral reactions.[3] The coaching thematizedthat thoughts, feelings and behavior influence each other and that psychological stress arises among other things due to irrational, distorted thought patterns. The ABC theory is introduced using an example (A triggering internal or external event, B evaluation of the event, C emotional and behavioral reaction). The participants then create their own examples. The following is worked out: It is not A that triggers
C, but B is important emotional reaction. Based on the models developed by the participants, various irrational beliefs are collected and discussed within the group. The ABC model is then supplemented with the new aspects of D (disputation) and E (new effect).

The second coaching session focused on stress management, resource activation, and relaxation strategies.The ABC model is also reintroduced again in order to identify and discuss negative self-verbalizations in and stressful situations. In the group, an exchange on functional methods for dealing with stress is encouraged. At the end, a short, psychoeducational unit is intended to encourage participants to practise relaxation techniques.Throughout both sessions different interactive media formats such as live quizzes and breakout rooms were used to enhance participation.
Coaching implementation

The coaching sessions were led by experienced clinicians and psychology students with a bachelor's degree. All coaches previously participated in two trial runs of the coaching sessions and received individual feedback before and during the intervention.

Participants of the coaching group were instructed to play the REThink app game and received two additional group coaching sessions in groups of 6 to 10 participants (duration 60 to 90 minutes). Participants were free to choose between online sessions or in-person sessions at the children's clinic, however, no in-person coachings were held due to low demand. During the intervention, coaching participants were reminded to participate via e-mail or phone one day before their sessions.

Supplementary analysis data

Supplementary Table 2: Normality tests for analysis of mean differences between groups

**Mean difference variable**	**p-Value Shapiro-Wilk**
Group A RS-13 scores pre-post	0.602
Group A RS-13 scores pre-follow-up	0.993
Group A CATS scores pre-post	0.052
Group A CATS scores pre-follow-up	0.489
Group B RS-13 scores pre-post	0.515
Group B RS-13 scores pre-follow-up	<0.001
Group B CATS scores pre-post	0.696
Group B CATS scores pre-follow-up	<0.001

Supplementary data on adherence

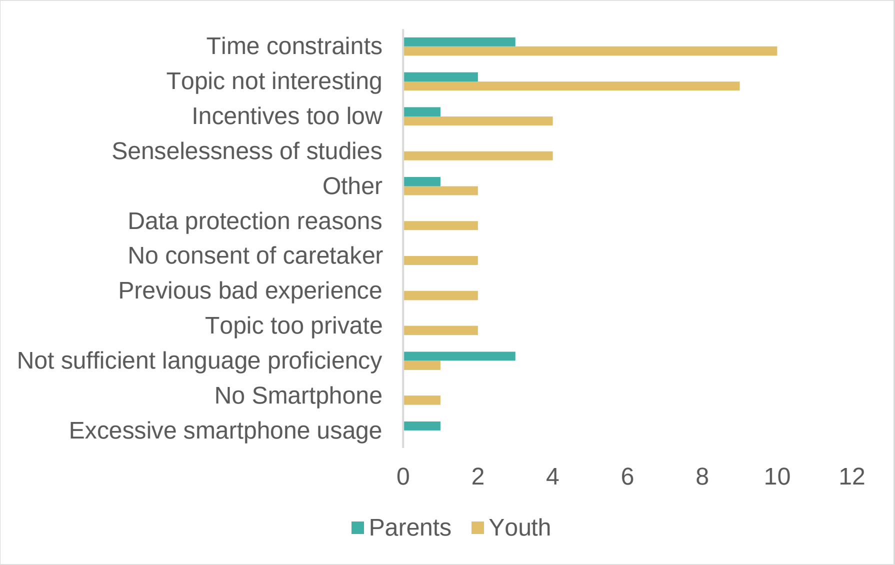

Supplementary Figure 1: Reasons for rejection by youths and parents (number of mentions)

Answers given n=21 youths and 8 parents (multiple answers possible).

Supplementary Table 3: Multivariate logistic regressions of coaching and app adherence on baseline characteristics

	Coaching compliance				***App compliance***			
	Multivariate		***Univariate***		***Multivariate***		***Univariate***	
	**Odds Ratio**	***p***	**Odds Ratio**	***p***	**Odds Ratio**	***p***	**Odds Ratio**	***p***
**Age (years)**	0.809	0.627	0.926	0.798	1.675	0.039	1.565	0.041
**Diabetes**	0.478	0.555	0.429	0.311	1.132	0.867	0.526	0.267
**SES**	1.996	0.032	1.872	0.03	1.096	0.474	1.082	0.505
**Group A**	-	-	-	-	0.208	0.208	0.533	0.277

SES=socioeconomic status. Diabetes= Type 1 diabetes mellitus.
Coaching compliance defined as having participated in both coaching sessions (only for Group REThink+Coaching). App compliance defined as having completed all seven levels.
Due to the disease variety and low disease frequencies type 1 diabetes mellitus (representing 55% of diseases) instead of disease was chosen as independent variable.

Supplementary data on app and coaching evaluation

Supplementary Table 4: App user age recommended by participants after app play

Recommended user age (in years)	Answers (in %)
<10	31
10-12	38
12-14	23
14-16	8
>16	0

Answers given n=39, percentage in relation to total number of mention.

Supplementary Table 5: Rating of difficulty of app

Rating	Answers (in %)
Very easy usage	31
Easy usage	38
Part/part	23
Difficult usage	8
Very difficult usage	0

Answers given n=39, percentage in relation to total number of mention.

Supplementary Table 6: Reasons for difficulties with app

Reasons	Answers (in %)
Description hard to understand	27.3
English language	45.4
Confusing navigation	0
Other: Information/texts changing too quickly	27.3

Answers given n=11, percentage in relation to total number of mention, multiple answers possible.

Supplementary data on media use

Supplementary Table 7: Frequency of average video game play

Frequency	Answers (in %)
Daily	27.5
3-4 x/Week	25.5
1-2x/Week	13.7
Rarely	23.5
Never	9.8

Answers given n=51, percentage in relation to total number of mentions.

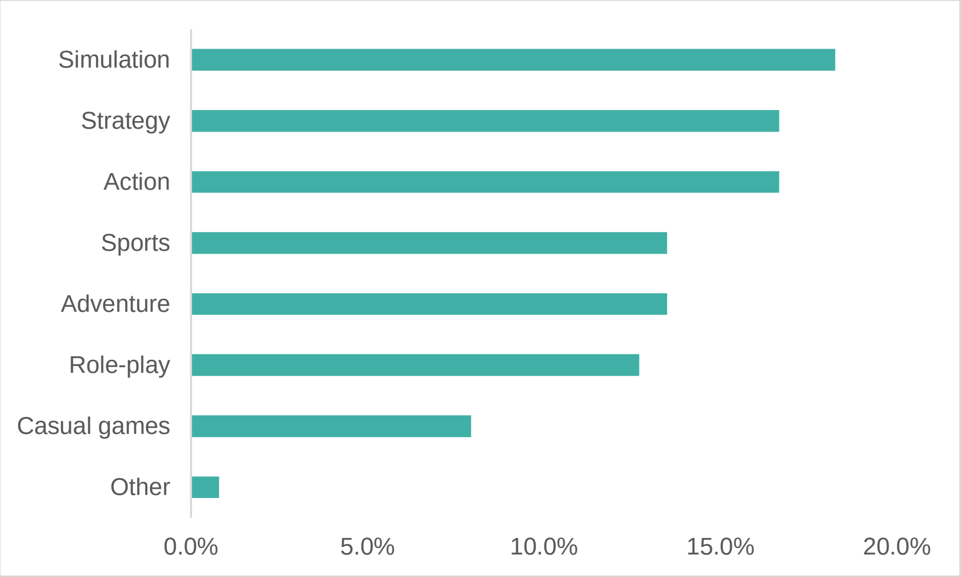

Supplementary Figure 2: Type of video games played (n answers in %)
Answers given n=126. Video game types given examples: Adventure (e.g. Pokemon Go, GTA), Action (e.g. Fortnite), Casual games (e.g. Fall Guys, Candy Crush), Role-play & online multiplayer (e.g. League of Legends), Strategy games (e.g. Clash of Clans, Among Us, Clash Royale), Simulation (e.g. Minecraft, Sims), Sports and racing (e.g. Fifa), Other (given answer: Fantasy games). Multiple answers possible.

Supplementary Table 8: Type of device used for playing video games

Type of device	Answers (in %)
Smartphone	38.9
Game console	32.0
Computer	22.2
Ipad/Tablet	6.9

Answers given n=42, percentage in relation to total number of mention, multiple answers possible.

Supplementary Table 9: Preferred player company for video game play

Player company	Answers (in %)
Friends	58.1
Alone	34.9
Siblings	7.0

Answers given n=43, percentage in relation to total number of mention.

Supplementary Table 10: Frequency media device use on a typical day (in % of answers)

	Ipad/Tablet	Console	Smartphone	Computer/Laptop
**Several times/d**	23.9	8.7	95.7	19.6
**1x/d**	8.7	6.5	2.2	8.7
**Several times/week**	6.5	10.9	2.2	10.9
**1-2x/week**	4.3	8.7	0.0	4.3
**Every other week**	10.9	13.0	0.0	15.2
**Rarely**	19.6	17.4	0.0	26.1
**Never**	26.1	34.8	0.0	15.2

Answers given n=46.

Supplementary Table 11: Duration of media device use on a typical day (in % of answers)

	Ipad/Tablet	Console	Smartphone	Computer/Laptop
**Never**	45.7	47.8	0.0	34.8
**1-29 min**	13.0	10.9	0.0	13.0
**30-59 min**	10.9	10.9	2.2	10.9
**1-2 h**	10.9	17.4	17.4	13.0
**2-3 h**	8.7	4.3	13.0	13.0
**3-4 h**	2.2	6.5	19.6	6.5
**4-5 h**	2.2	2.2	21.7	4.3
**>5h**	6.5	0.0	26.1	4.3

Answers given n=46, min=minutes, h=hours.

Supplementary Table 12: Availability of device at -home (in % of answers)

	Ipad/Tablet	Console	Smartphone	Computer/Laptop
**Not available**	10.9	26.1	0.0	10.9
**Belongs to siblings/parents**	43.5	19.6	0.0	37.0
**Own property**	45.7	54.3	100.0	52.2

Answers given n=46.

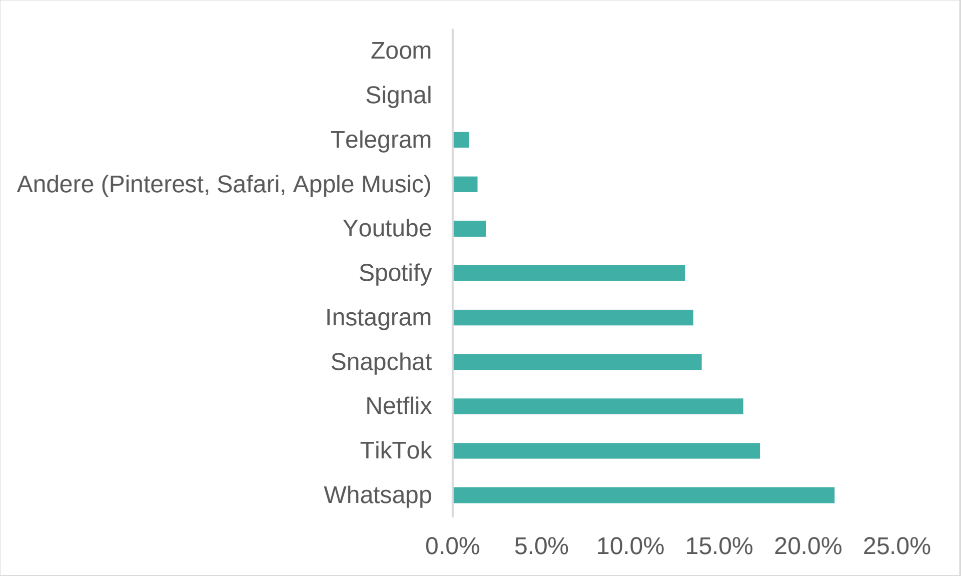

Supplementary Figure 3: Frequently used apps (number of mentions in %)

Answers given n=214, percentage in relation to total number of mention, multiple answers possible.

CONSORT 2010 checklist of information to include when reporting a pilot or feasibility trial[11]

Section/Topic	Item No	Checklist item	Reported on page No
**Title and abstract**			
	1a	Identification as a pilot or feasibility randomised trial in the title	1
	1b	Structured summary of pilot trial design, methods, results, and conclusions (for specific guidance see CONSORT abstract extension for pilot trials)	2
**Introduction**			
Background and objectives	2a	Scientific background and explanation of rationale for future definitive trial, and reasons for randomised pilot trial	3-4
	2b	Specific objectives or research questions for pilot trial	4
**Methods**			
Trial design	3a	Description of pilot trial design (such as parallel, factorial) including allocation ratio	5-6
	3b	Important changes to methods after pilot trial commencement (such as eligibility criteria), with reasons	n.a.
Participants	4a	Eligibility criteria for participants	4
	4b	Settings and locations where the data were collected	4
	4c	How participants were identified and consented	5
Interventions	5	The interventions for each group with sufficient details to allow replication, including how and when they were actually administered	6, Suppl. Inf.
Outcomes	6a	Completely defined prespecified assessments or measurements to address each pilot trial objective specified in 2b, including how and when they were assessed	4-6
	6b	Any changes to pilot trial assessments or measurements after the pilot trial commenced, with reasons	n.a.
	6c	If applicable, prespecified criteria used to judge whether, or how, to proceed with future definitive trial	4
Sample size	7a	Rationale for numbers in the pilot trial	4

	7b	When applicable, explanation of any interim analyses and stopping guidelines	n.a.
Randomisation:			
Sequence generation	8a	Method used to generate the random allocation sequence	5
	8b	Type of randomisation(s); details of any restriction (such as blocking and block size)	5
Allocation concealment mechanism	9	Mechanism used to implement the random allocation sequence (such as sequentially numbered containers), describing any steps taken to conceal the sequence until interventions were assigned	5
Implementation	10	Who generated the random allocation sequence, who enrolled participants, and who assigned participants to interventions	5, Suppl. Inf.
Blinding	11a	If done, who was blinded after assignment to interventions (for example, participants, care providers, those assessing outcomes) and how	n.a.
	11b	If relevant, description of the similarity of interventions	n.a.
Statistical methods	12	Methods used to address each pilot trial objective whether qualitative or quantitative	6
**Results**			
Participant flow (a diagram is strongly recommended)	13a	For each group, the numbers of participants who were approached and/or assessed for eligibility, randomly assigned, received intended treatment, and were assessed for each objective	7
	13b	For each group, losses and exclusions after randomisation, together with reasons	7-8
Recruitment	14a	Dates defining the periods of recruitment and follow-up	4, Suppl.Inf.
	14b	Why the pilot trial ended or was stopped	Suppl. Inf.
Baseline data	15	A table showing baseline demographic and clinical characteristics for each group	9-10
Numbers analysed	16	For each objective, number of participants (denominator) included in each analysis. If relevant, these numbers should be by randomised group	8,11
Outcomes and estimation	17	For each objective, results including expressions of uncertainty (such as 95% confidence interval) for any estimates. If relevant, these results should be by randomised group	10-13
Ancillary analyses	18	Results of any other analyses performed that could be used to inform the future definitive trial	7-9, Suppl.Inf.

Harms	19	All important harms or unintended effects in each group (for specific guidance see CONSORT for harms)	n.a.
	19a	If relevant, other important unintended consequences	n.a.
**Discussion**			
Limitations	20	Pilot trial limitations, addressing sources of potential bias and remaining uncertainty about feasibility	14-16
Generalisability	21	Generalisability (applicability) of pilot trial methods and findings to future definitive trial and other studies	14-16
Interpretation	22	Interpretation consistent with pilot trial objectives and findings, balancing potential benefits and harms, and considering other relevant evidence	14-16
	22a	Implications for progression from pilot to future definitive trial, including any proposed amendments	14-16
**Other information**			
Registration	23	Registration number for pilot trial and name of trial registry	1
Protocol	24	Where the pilot trial protocol can be accessed, if available	1
Funding	25	Sources of funding and other support (such as supply of drugs), role of funders	18
	26	Ethical approval or approval by research review committee, confirmed with reference number	18

Cochrane risk-of-bias tool for randomized trials (RoB 2) checklist [12]

**Ref or Label**		**Aim**	assignment to intervention (the 'intention-to-treat' effect)		
**Experimental**	REThink + Coaching	**Comparator**	REThink	**Source**	Trial protocol; Statistical analysis plan (SAP); Non-commercial trial registry record (e.g. ClinicalTrials.gov record); Research ethics application
**Outcome**	Feasibility, RS-13/CATS score				

Domain	Signalling question	Response	Comments
**Bias arising from the randomization process**	1.1 Was the allocation sequence random?	Y	The random allocation sequence list was stored in a sealed folder and consulted for allocation after successful recruitment.
	1.2 Was the allocation sequence concealed until participants were enrolled and assigned to interventions?	Y	
	1.3 Did baseline differences between intervention groups suggest a problem with the randomization process?	N	Chi-square and t-test analyses indicated no difference in baseline characteristics which suggests successful randomization.
	**Risk of bias judgement**	Low	
**Bias due to deviations from intended interventions**	2.1.Were participants aware of their assigned intervention during the trial?	Y	
	2.2.Were carers and people delivering the interventions aware of participants' assigned intervention during the trial?	Y	
	2.3. If Y/PY/NI to 2.1 or 2.2: Were there deviations from the intended intervention that arose because of the experimental context?	N	

	2.4 If Y/PY to 2.3: Were these deviations likely to have affected the outcome?	NA	
	2.5. If Y/PY/NI to 2.4: Were these deviations from intended intervention balanced between groups?	NA	
	2.6 Was an appropriate analysis used to estimate the effect of assignment to intervention?	Y	Data from all randomized participants were analyzed (intention-to-treat-collective) (descriptive analysis, Wilcoxon signed-rank test, ANOVA, logistic regressions)
	2.7 If N/PN/NI to 2.6: Was there potential for a substantial impact (on the result) of the failure to analyse participants in the group to which they were randomized?	NA	
	**Risk of bias judgement**	Low	
**Bias due to missing outcome data**	3.1 Were data for this outcome available for all, or nearly all, participants randomized?	Y	Primary endpoint was feasibility which included all randomized participants. Outcome data for RS-13/CATS-score (secondary endpoints) available for 92% of randomized participants before intervention. 76% of rand. participants completed post-intervention assessment, 86% of rand. participants completed follow-up assessment).
	3.2 If N/PN/NI to 3.1: Is there evidence that result was not biased by missing outcome data?	NA	
	3.3 If N/PN to 3.2: Could missingness in the outcome depend on its true value?	NA	
	3.4 If Y/PY/NI to 3.3: Is it likely that missingness in the outcome depended on its true value?	NA	
	**Risk of bias judgement**	Low	
**Bias in measurement**	4.1 Was the method of measuring the outcome inappropriate?	N	

**of the outcome**	4.2 Could measurement or ascertainment of the outcome have differed between intervention groups?	N	All participants received the same questionnaire within the same time frame.
	4.3 Were outcome assessors aware of the intervention received by study participants?	Y	The study team members who send out the post-intervention questionnaire links were ware of the intervention group of the participants.
	4.4 If Y/PY/NI to 4.3: Could assessment of the outcome have been influenced by knowledge of intervention received?	N	The participants received the same questionnaire link regardless of their intervention group. The online questionnaire was completed without attendance of the investigators (e.g. at home) and was independent from investigator assessment.
	4.5 If Y/PY/NI to 4.4: Is it likely that assessment of the outcome was influenced by knowledge of intervention received?	NA	
	**Risk of bias judgement**	Low	
**Bias in selection of the reported result**	5.1 Were the data that produced this result analysed in accordance with a pre-specified analysis plan that was finalized before unblinded outcome data were available for analysis?	Y	The analysis plan was pre-specified in the study protocol and study registry.
	5.2 ... multiple eligible outcome measurements (e.g. scales, definitions, time points) within the outcome domain?	N	
	5.3 ... multiple eligible analyses of the data?	N	All analyses, which were performed according to the analysis plan, were published in the manuscript or supplementary information.
	**Risk of bias judgement**	Low	

**Overall bias**	**Risk of bias judgement**	Low	

References

1. GraphPad. Randomly assign subjects to treatment groups. In: GraphPad; 2022: DOI: DO
2. Leiner DJSSVCsAahwsd. In: DOI: DO
3. Ellis A, Ellis DJ. Rational emotive behavior therapy. In: Psychotherapy theories and techniques: A reader. Washington, DC, US: American Psychological Association; 2014: 289-298. DOI: 10.1037/14295-031
4. David O, Predatu R, Roxana C. A pilot study of the REThink online video game applied for coaching emotional understanding in children and adolescents in the therapeutic video game environment: The Feeling Better resources game. Journal of Evidence-Based Psychotherapies 2018; 18: 57-68. DOI: 10.24193/jebp.2018.1.5
5. David OA, Cardoș RAI, Lupu V. Promoting mental health in children and adolescents by using the REThink online therapeutic game: using empirically tested psychological contents. Revista de Neurologie şi Psihiatrie a Copilului şi Adolescentului din România 2016; 22.
6. David OA, Magurean S, Tomoiagă C. Do Improvements in Therapeutic Game-Based Skills Transfer to Real Life Improvements in Children's Emotion-Regulation Abilities and Mental Health? A Pilot Study That Offers Preliminary Validity of the REThink In-game Performance Scoring. Frontiers in psychiatry 2022; 13. DOI: 10.3389/fpsyt.2022.828481
7. David OA, Cardos RAI, Matu S. Is REThink therapeutic game effective in preventing emotional disorders in children and adolescents? Outcomes of a randomized clinical trial. Eur Child Adolesc Psychiatry 2019; 28: 111-122. DOI: 10.1007/s00787-018-1192-2
8. David OA, Fodor LA. Preventing mental illness in children that experienced maltreatment the efficacy of REThink online therapeutic game. npj Digital Medicine 2023; 6: 106. DOI: 10.1038/s41746-023-00849-0
9. David OA, Fodor LA. Are gains in emotional symptoms and emotion-regulation competencies after the REThink therapeutic game maintained in the long run? A 6-month follow-up. European Child & Adolescent Psychiatry 2023; 32: 1853-1862. DOI: 10.1007/s00787-022-02002-w
10. Bischops AC, Reinauer C, Pischke C et al. Strengthening the Resilience of Children and Adolescents during a Pandemic: A Scoping Review on Eligible Interventions. Klin Padiatr 2022.
11. Eldridge SM CC, Campbell MJ, Bond CM, Hopewell S, Thabane L, et al. CONSORT 2010 statement: extension to randomised pilot and feasibility trials. . BMJ 2016;355 2016.
12. RoB2 Development Group. Revised Cochrane risk-of-bias tool for randomized trials (RoB 2). In: Julian PT Higgins JS, Matthew J Page, Jonathan AC Sterne, ed 2019: DOI: DO
